# Acceptability and Feasibility of a Telehealth Intervention for Sexually Transmitted Infection Testing Among Male Couples: Protocol for a Pilot Study

**DOI:** 10.2196/14481

**Published:** 2019-10-01

**Authors:** Stephen Sullivan, Patrick Sullivan, Rob Stephenson

**Affiliations:** 1 Center for Sexuality and Health Disparities University of Michigan School of Nursing Ann Arbor, MI United States; 2 Rollins School of Public Health Department of Epidemiology Emory University Atlanta, GA United States; 3 Department of Systems, Population and Leadership University of Michigan School of Nursing Ann Arbor, MI United States

**Keywords:** sexually transmitted diseases, telemedicine, sexual and gender minorities

## Abstract

**Background:**

Gay, bisexual, and other men who have sex with men (MSM) are at elevated risk for acquiring sexually transmitted infections (STIs) in the United States, especially chlamydia and gonorrhea. While research has indicated main partners over casual partners may play a central role in STI risk, the frequency of STI screening among MSM couples is particularly low. Self-sample collection for chlamydia and gonorrhea screening has been shown to be highly accurate, and at-home STI testing has been shown to be highly acceptable among diverse populations. However, there is little research exploring the feasibility and acceptability of at-home chlamydia and gonorrhea screening among MSM couples. Our pilot study aims to help evaluate the viability of this screening modality as an intervention tool for MSM couples

**Objective:**

The objective of this study was to assess the feasibility and acceptability of an at-home chlamydia and gonorrhea sample collection and remote lab testing program among a sample of 50 MSM couples living in the United States.

**Methods:**

This pilot study enrolled 50 MSM couples, ranging from 18-40 years old and living in the United States, who participated in a larger at-home HIV testing randomized controlled trial. Participating couples completed a pretest instructional video call and then had the option of completing at-home sample collection across three bodily sites (rectal swab, pharyngeal swab, and urine sample) for remote chlamydia and gonorrhea lab testing. For participants who completed any sample collection, they received their results via a posttest video call. All participants completed an online survey examining satisfaction and acceptability of the home testing process, experience with logistics, willingness to test at home in the future, recent sexual risk behavior, STI testing history, and linkage to care. A subset of 10 couples completed an in-depth interview about their attitudes towards the sample collection process, different decisions they made while collecting their samples, and their experience accessing treatment (for those who received a positive result).

**Results:**

Recruitment began in September 2017, and as of March 2019 a total of 50 couples have been enrolled. Overall, 49/50 couples have returned their samples and completed the posttest delivery call, and 10 in-depth interviews have been completed and transcribed.

**Conclusions:**

Screening MSM couples at home for chlamydia and gonorrhea and providing video-facilitated results delivery may offer a tailored approach to address the increasing prevalence of these STIs. By collecting data on how MSM couples experience at-home STI screening, this project will provide valuable insight into the utility of such a service delivery program to public health interventionists and researchers alike.

**International Registered Report Identifier (IRRID):**

DERR1-10.2196/14481

## Introduction

In the United States, gay, bisexual, and other men who have sex with men (MSM) are at elevated risk for acquiring sexually transmitted infections (STIs) relative to heterosexual men and women [1-4]. Although women in the United States experience a higher burden of chlamydia than men, increased screening over time has shown the rate of infection among men increased by almost 40% between 2013-2017, a rise primarily attributed to rising incidents of infections among MSM [5]. Across 30 STI clinics within 10 US STI surveillance districts, the median site-specific chlamydia prevalence for MSM was 4.8% for urethral specimens, and 16.8% for rectal specimens in 2017 [5]. Among those same clinics, the median site-specific gonorrhea prevalence for MSM was 8.5% for urethral specimens, 14.7% for rectal specimens, and 13.4% for pharyngeal specimens in 2017 [5]. This is particularly troubling given the appearance of increasingly prevalent strains of treatment-resistant gonorrhea. Using gonorrhea case data derived from 27 US state or city health departments from 2011-2016, the Gonococcal Isolate Surveillance Project found that isolates from MSM were significantly more likely to show signs of antimicrobial resistance than specimens from heterosexual men [5]. With such high rates of chlamydia and treatment-resistant gonorrhea, improving routine screening among this population has become a key public health priority.

Counter to past conceptualizations that posited casual sex partners were central to STI risk, recent modeling analyses indicate that between one- and two-thirds of new HIV infections among MSM are attributable to main sexual partners [6,7]. Although these findings are specific to HIV, they remain relevant to chlamydia and gonorrhea due to the high comorbidity of these STIs and their similar transmission pathways [8]. Infections derived from main partners are driven by MSM being more likely to have anal sex, being less likely to use condoms with their main partners compared to casual partners [7,9-11], and tending to have more overall sex acts per year with main partners [7]. To compound this elevated risk context, MSM with main partners report lower levels of routine HIV testing than single MSM [12,13]. While data on the prevalence of STI testing among MSM couples is limited, Mitchell and Petroll found that, on average among 361 MSM couples in the United States at the time of the survey, they reported testing for other STIs, besides HIV, about 19.1 months ago [14]. These high rates of HIV transmission, sexual risk behavior, and low frequency of HIV or STI screening suggest that MSM couples may be at particular risk for undiagnosed STIs such as chlamydia and gonorrhea.

To curb the STI epidemic in the United States, experts have increasingly called for more efficient deployment of self-testing modalities among populations at risk [15]. Calls to increase self-testing are partially due to home testing technology demonstrating similar effectiveness in identifying and treating cases of STIs relative to clinic-based testing strategies. A Cochrane review of 10 clinical trials that compared home-based chlamydia or gonorrhea testing to clinic-based testing found that home-based testing achieved higher screening uptake among participants and resulted in similar levels of treatment [16]. Another meta-analysis of self-collected chlamydia or gonorrhea samples versus clinician-collected samples found similarly high sensitivity and specificity of self-collected swabs and urine samples [17]. Beyond effectiveness, at-home STI testing is considered acceptable across diverse populations. A systematic review of patient experiences when self-testing for curable STIs found, across 36 studies, that 85% of participants reported self-sampling as being acceptable, and across 28 studies, 88% of participants reported it was easy to use while only 13% reported any pain or discomfort [18].

Although at-home STI testing has been successfully administered to single MSM [19-21], to our knowledge no such data exists on the acceptability and feasibility of at-home STI testing among MSM couples. Across 15 couple-based interventions that focused on at least one HIV prevention outcome (ie, sex or drug use behavior, HIV or STI testing, HIV treatment uptake, and medication adherence), a recent meta-analysis found that these dyadic interventions produced significantly higher changes in sexual risk, HIV testing, and Nevirapine uptake relative to individual intervention comparison groups [22]. This evidence suggests that interventions designed for couples operate on behavioral change outcomes in significantly unique ways relative to interventions designed for individuals. Therefore, individual MSM data on the acceptability and feasibility of an intervention, such as STI screening, may not be translatable to how MSM couples would experience the same intervention as a dyad. Since there were only two studies in the meta-analysis examining STI testing (both of which were conducted among heterosexual couples), there was also insufficient evidence to examine how couple level interventions may produce different effects in STI screening compared to individualistic approaches [22]. The dearth of couples’ STI testing studies signals the need for more research in this area, particularly among MSM couples. This study begins to address this gap by developing and piloting an at-home STI testing protocol specifically designed for MSM couples.

The home environment may be uniquely suited to reach at-risk MSM couples for STI screening. Piloting research on how male couples experience at-home sample collection and remote lab testing may offer critical data in evaluating the viability of this screening modality as an intervention tool. This paper describes the protocol for a pilot study of at-home chlamydia and gonorrhea testing and remote video call results delivery for MSM couples living in the United States and aged between 18-40 years old. The procedures described below have been reviewed and approved by the Institutional Review Board at the University of Michigan in Ann Arbor (HUM00131366) and have been deemed to pose no more than minimal risk to study participants.

## Methods

### Study Overview

The Project Nexus STI At-home Testing Pilot Study is an exploratory project of 50 male same-sex couples, aged 18 to 40 years old and living in the United States, who recently completed a larger clinical trial of remote couples’ HIV testing and counseling via remote video call [23]. The age range restriction was developed in order to better align with the younger trend of chlamydia and gonorrhea diagnoses in the MSM community [5]. To be eligible, couples needed to be HIV-negative by the end of the original trial, report still being in a relationship with the partner they started the original study with, and both had to express their interest to participate to study staff individually. Eligible couples scheduled an introductory Health Insurance Portability and Accountability Act (HIPAA)-compliant video call with study staff, who reviewed the informed consent document, obtained their verbal consent to participate, and demonstrated how to use the at-home chlamydia and gonorrhea sample collection kit materials. After completing the call, each partner was sent a discreet box containing instructions and supplies to self-collect a pharyngeal swab, a rectal swab, and a urine sample. For those who decided to collect and return their samples, they were screened for chlamydia and gonorrhea. Testing across these three sites is of importance because, until recently, testing protocols for gonorrhea and chlamydia among MSM mainly focused on detecting urethral infections, which are more likely to be symptomatic than pharyngeal and rectal infections [24]. Once study staff received each partner’s multi-site test results from the processing laboratory, the couple scheduled another video call with study staff to go over their test results and receive tailored risk reduction counseling. Upon receiving their test results via video conferencing, each partner was sent an incentivized online survey, to complete individually, that aimed to assess recent sexual risk behavior, STI testing history outside of the study, and acceptability of the at-home testing procedures. Lastly, we conducted 10 in-depth interviews with 10 couples to explore any potential barriers or facilitators to their experience collecting their samples at home and receiving their results through video conferencing.

### Participant Recruitment

The STI at-home testing pilot recruited and enrolled 50 male same-sex couples, aged 18-40 years old, who were sero-concordant, HIV-negative, and resided in the United States. Participants were recruited from past participants who completed the original at-home HIV testing study and who, by the last follow-up survey, reported being HIV-negative and were still together with their romantic partner. The original trial recruited male couples across the United States via online advertisements placed on Facebook, Instagram, Scruff, Grindr, and Poz Magazine, and through email referrals sent to past respondents of the Emory-based American Men’s Internet Survey. Study staff reviewed participant data in the original study to compile a list of eligible couples to invite to participate. Couples were contacted regarding their eligibility via email, phone call, or text message, depending on their contact preferences using standardized scripts. Both partners had to individually express interest in the study before continuing forward.

### Study Procedures

#### Pretest Instructional Call

If both partners of an eligible couple communicated interest in participating in the pilot project individually, study staff scheduled a preliminary video call with the couple that lasted approximately 15 minutes. Before the scheduled call, the eligible couple was sent the informed consent document for their review. This video call was conducted using a HIPAA-compliant video call service called VSee. During the preliminary video call, study staff went over the informed consent form, clarified any questions the couple had, and asked if they chose to provide consent to join the pilot project. While reviewing the consent form, study staff also discussed the risk of potentially feeling coerced into participation by their partner. Anyone experiencing coercion from their partner was encouraged to contact study staff privately and those couples would be independently offered the at-home testing kits as well as a list of local STI screening centers if they preferred alternate mode of testing, but they would not be included in the study sample. Men who did not consent, or whose partner did not consent, were thanked for their time but did not move forward in the study. If both partners agreed, the staff member then went over the contents of the three-site, at-home gonorrhea and chlamydia test kit, and explained how they would use each element. The instructions included going over the urine sampling procedure, the rectal swab sampling method, and the pharyngeal swab process. Study staff screen-shared images of the appropriate sampling procedure and physically demonstrated with the self-collection supplies the effective techniques following laboratory guidelines. Study staff also went over frequently asked questions tailored to the behaviors of MSM, such as avoiding rectal douching before collecting the rectal sample. After the couple had become familiar with the test kit and communicated understanding about how they would collect their test samples, study staff then confirmed the shipping address where they would like to have their test kit sent. Afterward, study staff conducting the call filled out a case report form as a fidelity measure documenting the couples’ verbal consent and the instructions provided.

#### At-Home Sample Collection

#### Overview

Once study staff confirmed a couple’s shipping address, they sent one package containing two sample collection kits, one for each partner. The box was packaged discreetly, with no identifiable information regarding the study on the exterior. Each sample collection kit had the corresponding partner’s first name labeled on the exterior to indicate which kit was assigned to them. All test sample materials returned for lab processing were only identified by a randomly generated number separate from their study identifier to blind the processing laboratory to the identities of participants. Package instructions not only detailed the sample collection procedures covered in the pretest video call but urged participants to return their test samples as soon as possible. Couples were informed they could either collect the samples separately or together, depending on their preference. Couples could also choose to return some, or none of their samples for chlamydia and gonorrhea testing. The package instructions included detailed written descriptions of self-collection techniques for their pharyngeal, urine, and rectal samples, alongside color illustrations for further guidance.

#### Pharyngeal Swab Self-Collection

Each partner was instructed to wash their hands thoroughly prior to collection. Then, they unscrewed the top of a transport tube and opened the sterile packaging of a cotton swab stick. With the swab stick in hand, each partner swabbed the back of their throat on either side where their tonsils were located. Upon completion, they inserted the cotton swab end of the stick into the transport tube and broke off the top half, leaving the swab inside. Upon tightly screwing on the top of the transport tube and placing it into a provided biohazard bag, each partner could throw away the leftover materials.

#### Urine Sample Self-Collection

Each partner was instructed to wait at least one hour since they last urinated before collecting their urine sample. Once the proper amount of time had elapsed, participants again washed their hands. Then, they unscrewed the sealed lid of a 30-ounce specimen cup and collected the first initial stream of urine into the cup, filling it to a demarcated line at 5 ounces. After washing their hands again, participants extracted some of the urine using a pipette, unscrewed the lid of another transport tube, and filled the tube with urine between the minimum and maximum fields on the tube. Finally, each partner tightly screwed the top on the transport tube and inserted it into the same biohazard bag. The pipette, specimen cup, and instructions could then be thrown away in the garbage.

#### Rectal Swab Self-Collection

Prior to self-collection, participants needed to wash their hands a final time and then unscrew the cap of the last transport tube. Like the pharyngeal swab, they opened the sterile paper packaging to remove the cotton swab stick. Each partner then inserted the swab into his rectum an inch and a half, using provided lubricant as needed. With the swab stick inside at the right depth, they rotated the swab gently in a circular motion several times and then withdrew. Placing the cotton end of the swab into the transport tube, they broke off the stick portion, leaving the cotton end inside. As a final step, each partner screwed on the top of the tube and placed it in the biohazard bag with the rest of their samples. The remaining materials could then be thrown away.

#### Sample Return

With all three samples in the biohazard bag, each partner then sealed the bag and wrote in the current date on a separate lab information form. Both the biohazard bag and the completed form were inserted into a bubble wrap mailer with a prepaid FedEx return shipping label affixed to the front. While the swab and urine samples are considered stable between 35°F-86°F for up to 30 days, the return packaging materials were designed to help prevent sample degradation during transit. The return shipment type was FedEx standard overnight delivery so that the samples could arrive at the processing laboratory by the next business day and minimize exposure to unideal conditions. The biohazard bag was a ThermoSafe specimen transport bag with absorbent sheets to protect against spills, and the return shipment package was an opaque bubble wrap mailer to reduce ultraviolet exposure and physical damage during shipment. The return shipment packaging was discreet and mentioned nothing regarding the nature of the study nor any identifiable information traceable to either partner. After sealing their return shipment package, each partner was able to either schedule for FedEx to pick up the package or find a FedEx store, or drop-off box, to leave it at to be shipped. Collecting and returning specimens was not incentivized and participants could continue in the study without collecting them. All samples were sent to the Emory University Clinical Virology Laboratory to be tested using the US Food and Drug Administration approved Abbot real-time chlamydia trachomatis and Neisseria gonorrhea polymerase chain reaction assay, which detects a region of the cryptic plasmid DNA of Chlamydia trachomatis and a region of the Opa gene of Neisseria gonorrhea [25]. For chlamydia trachomatis the assay’s sensitivity=92.4% and its specificity=99.2%, and for Neisseria gonorrhea the assay’s sensitivity=96.9% and its specificity=99.7% [25]. Results from the processing lab were shared with study staff through a HIPAA-compliant cloud storage service called Box. Only institutional review board–approved study staff were able to link test results to any participant via the randomly assigned sample identifier and the study identifier.

#### Posttest Results Delivery Call

Once study staff received both partners’ chlamydia and gonorrhea test results, they contacted the couple to schedule another video call via VSee that would last approximately 20 minutes. The purpose of the call was to deliver each partner’s test results, provide linkage to care if needed, and create a prevention plan tailored to the couple’s sexual behavior and risk. Study staff who facilitated these calls were certified in HIV counseling, testing, and referral, and trained in couples’ HIV testing and counseling. During the call, both partners needed to verbally consent to receive their test results together or opt in to receive their results separately over two video calls scheduled at different times. Both partners also needed to agree to mutual confidentiality and disclosure (ie, agreeing they would not divulge their partner’s test result to someone outside of their relationship without their partner’s prior consent). They could also refuse to receive their results entirely and continue with the rest of the study. If both partners consented and agreed to the confidentiality and disclosure stipulation, the study team member would start by reiterating what samples were collected and the chlamydia and gonorrhea results that were possible (eg, having a positive result at one area of the body but not anywhere else). Afterward, study staff screen shared a document containing each partner’s test results across each of the three bodily sites and discussed each one. Upon receiving their results, study staff facilitated a conversation around sexual risk behavior, STI prevention, and screening. After completion, study staff conducting the call filled out a case report form as a fidelity measure, documenting the couples’ consent, results, and prevention plan.

When participants received a positive chlamydia or gonorrhea result, study staff sent them an email of local STI treatment clinic referrals within 48 hours of the result delivery video call. During the call, study staff requested the location where the participants would like to receive STI clinic information from and discussed the importance of getting treatment. Negative partners of an individual with a positive test result were also encouraged to screen again and were given the option of receiving local STI screening sites in their area. Resources were compiled using the Centers for Disease Control (CDC) testing site locator and the United Way 211 organization database. Study staff followed up with any individual who received a positive test result 14 days after the result delivery call to assess receipt of treatment over the phone and help troubleshoot any issues encountered. A case report form was filled out after this follow-up call to document treatment status or reasons for not seeking treatment. Participants who received an invalid chlamydia or gonorrhea result for any one, or all, their samples were offered a list of STI testing sites in a location of their choice, but they were not provided another at-home chlamydia and gonorrhea sample collection kit through the study.

#### Acceptability Survey

After receiving their results or refusing to take part in the at-home testing portion of the study, participants were sent an email with a hyperlink to a secure online survey programmed in Qualtrics (Qualtrics XM, Seattle). The survey included questions regarding satisfaction and acceptability of the home testing process, experience with logistics (ie, the clarity of the pretest instructions), willingness to test at home in the future, recent sexual risk behavior, STI testing history, and linkage to care. Satisfaction, acceptability, and willingness measures were adapted from the Client Satisfaction Questionnaire [26] alongside those derived from other studies of at-home HIV testing trials [23,27]. Sexual behavior was assessed by using measures adapted from the National HIV Behavioral Surveillance inventory [28]. Participants were asked to estimate the number of anal sex encounters they had with their primary partner and any outside partners, the number of those encounters where condoms were used, and the number of times where they were the insertive or receptive sexual partner. STI screening questions and linkage to care items were based on the CDC’s STI screening and treatment guidelines for chlamydia and gonorrhea [29]. Participants were sent a reminder to complete the survey via their preferred contact method two weeks after receiving it, and again four weeks later. After those two reminders they were not contacted again.

#### In-Depth Interview

A subset of 10 couples who completed the testing portion of the study were invited via email to participate in an in-depth interview conducted remotely via VSee video call that lasted approximately 30 minutes. Study team members recruited participants through purposive sampling to help obtain interviews with five couples where at least one partner received a positive test result and five couples where both partners received negative results, as well as to have at least 10/20 participants identify as nonwhite. During the interview, couples were asked about their attitudes towards the sample collection process, different decisions they made while collecting their samples (ie, collecting their samples together or separately), and their experience accessing treatment (for those who received a positive result). Verbal consent to have the audio of the interview recorded and transcribed for future analysis was required from both partners for the interview to proceed. Upon receiving an initial invitation to participate in the interview via their preferred contact method, unresponsive couples were invited once more two weeks later. After those two invitations they were not contacted again.

### Analysis

Descriptive statistics will be used to quantify characteristics of participating couples, such as relationship status, STI testing history, and recent sexual behavior, using Stata version 14 [30]. Descriptive statistics will also be used to assess overall satisfaction and acceptability of the home sample collection procedure and delivery of results among participants. Proportions of each sample type (ie, rectal, pharyngeal, urine) returned and the adequacy of the specimen collection for chlamydia and gonorrhea testing will be calculated. Linkage to care will be analyzed through aggregated counts of follow-up interactions detailing receipt of treatment post-result delivery. Chi-square tests will be used to assess any significant differences in positive versus negative experiences of the intervention by various demographic variables (ie, race, ethnicity, age, relationship type).

In-depth interviews will be transcribed and double checked for any discrepancies with the original audio files. All identifiable information, such as names and locations, will be removed before analysis. Verified transcripts will undergo a deductive coding strategy wherein a code hierarchy of key information (ie, attitudes toward the sample collection procedure, reasons for testing together or separately, experiences with finding treatment) will be developed by the research team and used by analysts to systematically code the qualitative data [31]. Analysts will meet regularly during the coding phase to discuss and reconcile coding inconsistencies, as well as revisit the code structure for possible revision and additions if there are described experiences not captured by the original schema. After the coding phase has been completed, thematic analysis will be conducted on extracted quotes by code area to pull overarching themes across the dataset.

### Incentives

All participants received a $50 Amazon electronic gift card via email for completing the acceptability survey and an additional $30 Amazon electronic gift card for completing the in-depth interview. Returning samples to be tested for chlamydia and gonorrhea or completing the post-test results delivery call were not incentivized study activities.

## Results

Recruitment began in September 2017 and ended in October 2018. A total of 75 couples were screened and deemed eligible and then invited to participate in the pilot study. Nineteen couples were either uninterested or did not reply to the invitation, 5 couples reported no longer being together, and 1 couple dropped out after initial enrollment. A total of 50 couples were enrolled, with 49 returning their samples and completing the posttest delivery call. Nine individuals tested positive for chlamydia or gonorrhea, and all successfully obtained treatment at the 14-day follow-up call. Ten in-depth interviews were completed and transcribed. Analysis of the study results is currently underway.

## Discussion

The Project Nexus STI At-Home Testing Pilot Study aims to evaluate preliminary acceptability of at-home chlamydia and gonorrhea self-collection and remote, video-facilitated results delivery among MSM couples living in the United States. Being able to test and receive STI results at home with a primary partner may help reduce barriers to screening and facilitate tailored conversations around sexual health for each couple’s specific circumstances. Because current CDC guidelines for expedited partner therapy (providing antibiotic treatment for chlamydia or gonorrhea to an external sexual partner who has yet to be tested) only pertain to heterosexual cases [32], having MSM couples screen together for these STIs may help ensure both partners can access treatment or retesting without solely relying on partner notification. By exploring the feasibility of at-home chlamydia and gonorrhea screening among MSM couples, our pilot may identify new avenues of research to help curb the STI epidemics among this highly affected population.

Because our pilot project derived its sample from a larger study of at-home HIV testing, the generalizability of our findings will be limited to this context. Our participants fully completed participating in a research trial with a 6-month follow-up period, so each partner’s ability to coordinate with one another on study-related tasks may be systematically different than couples who did not fully complete the original study. Moreover, couples who were still in a relationship by the time they were invited to participate in the pilot study may be demographically unique from those who were no longer together by the time of invitation. During analysis, we will compare our sample to those who were uninterested in participation, and who reported no longer being together, to determine any statistically significant differences. To our knowledge, no prior research has attempted to survey how MSM couples experience at-home STI screening, and with this context we believe our research will still be able to provide a useful base for future inquiry. Additionally, by allowing couples to opt out of the STI screening portion of the study, our ability to assess the acceptability of the sample collection materials and linkage to care could have been reduced. However, we believe that providing this option offered meaningful data on the willingness and interest MSM couples may have in these services. Lastly, we recognize the possibility of conflict that may arise when couples receive sensitive STI test results together. To minimize this potential adverse implication, we allowed partners the option to receive their results individually and we monitored relationship dissolution throughout the project period. However, we did not collect data on other indicators of relationship conflict, such as intimate partner violence or interpersonal stress, so our ability to assess conflict stemming from the at-home STI testing experience is limited to reports of relationship dissolution.

Despite these limitations, screening MSM couples at-home for chlamydia and gonorrhea and providing video-facilitated results delivery may offer a tailored approach to addressing the increasing prevalence of these STIs. By collecting data on how MSM couples experience at-home STI screening, this project will provide valuable insight into the utility of such a service delivery program to public health interventionists and researchers alike.

## Abbreviations

CDC: Centers for Disease Control
HIPAA: Health Insurance Portability and Accountability Act
MSM: men who have sex with men
STI: sexually transmitted infection
